# The Power of Licorice (*Radix glycyrrhizae*) to Improve Oral Health: A Comprehensive Review of Its Pharmacological Properties and Clinical Implications

**DOI:** 10.3390/healthcare11212887

**Published:** 2023-11-02

**Authors:** Hebah AlDehlawi, Ahoud Jazzar

**Affiliations:** Department of Oral Diagnostic Sciences, Faculty of Dentistry, King Abdulaziz University, Jeddah 21589, Saudi Arabia; ojazzar@kau.edu.sa

**Keywords:** bioactive compounds, candidiasis, dental caries, *Glycyrrhiza glabra*, halitosis, herbal medicine, licorice (*Radix glycyrrhizae*), periodontitis, recurrent aphthous ulcer

## Abstract

Licorice (*Radix glycyrrhizae*) is a plant root extract widely used in various applications, including cosmetics, food supplements, and traditional medicine. It has a long history of medicinal use in different cultures due to its diverse pharmacological properties. Licorice has traditionally been used for treating gastrointestinal problems, respiratory infections, cough, bronchitis, arthritis, and skin conditions. In recent years, the potential therapeutic benefits of licorice for oral health have gained significant interest. This paper aims to provide a comprehensive review of the effects of licorice extracts and their bioactive components on common oral diseases such as dental caries, periodontitis, halitosis, candidiasis, and recurrent aphthous ulcers. The chemical composition of licorice has shown the presence of several bioactive compounds such as glycyrrhizin, glabridin, isoliquiritigenin (ISL), and licochalcone exhibiting various pharmacological activities, including anti-inflammatory, antimicrobial, antioxidative, and immunomodulatory effects. Interestingly, in certain patients, licorice has shown a promising potential to inhibit the spread of viruses, prevent biofilm formation, reduce inflammation, boost immune responses, alleviate pain, and exert antioxidative effects. In this review, we provide a brief overview of the current understanding of licorice’s therapeutic benefits in the treatment of oral ailments, emphasising its potential as an alternative treatment option for oral diseases. Further research is warranted to explore its efficacy, safety, and clinical applications using placebo-controlled clinical trials.

## 1. Introduction

Licorice root (*Radix glycyrrhizae*) is obtained from the roots of *Glycyrrhiza glabra* L. (*G. glabra*), a small, flowering, bushy perennial plant of the family Fabaceae that is native to Western Asia, North Africa, and Southern Europe [1]. It is also sourced from *Glycyrrhiza uralensis* Fisch, referred to as Chinese licorice, which is a perennial herb of the family Fabaceae native to Asia [2]. The roots of these plants are harvested, dried, and then processed to produce licorice root extract, which is used in a variety of applications, including cosmetics, foods, tobacco, and traditional and herbal medicine [3]. The importance of medicinal plants is acknowledged on a global scale. They play a crucial role in the lives of rural residents, particularly in the remote parts of nations with few modern health services. Over 70,000 plant species are known to be used for therapeutic reasons [4]. Many studies have investigated the preservation of traditional knowledge and aimed to accelerate the development of new pharmaceuticals since the biodiversity of natural plants in various places offers quick, affordable, and ample alternative supplies for local health care [5]. Cosmetic products utilise licorice root extract due to its anti-inflammatory and skin-soothing properties [6]. In traditional Chinese medicine, “nine out of ten formulae contain licorice”; it is considered one of the essential herbal medications [7]. Licorice root has traditionally been used in herbal medicine to treat gastrointestinal problems [8], such as gastritis and peptic ulcers [9]. It is still commonly recommended for respiratory infections, coughs, bronchitis [10], and arthritis. Additionally, it is applied topically to manage skin conditions like eczema and psoriasis, as well as enhance wound healing [11,12,13]. Recently, the use of licorice in the treatment and management of oral diseases has been recognised [14]. Oral health is an important aspect of overall health and wellbeing. Maintaining good oral health involves maintaining healthy teeth, gums, and oral tissues, which can contribute to preventing oral diseases. Common oro-dental conditions treated or managed by licorice include dental caries, periodontitis, halitosis, candidiasis, and recurrent aphthous ulcers. This paper aims to provide a review of the potential therapeutic benefits of licorice extracts and their bioactive components. This study intends to shed light on the wide variety of therapeutic possibilities that licorice has by exploring the most recent scientific research and upcoming results. It underscores the need for more investigation, including placebo-controlled clinical studies, to thoroughly evaluate the effectiveness, safety, and therapeutic uses of licorice as a viable alternative therapy option for oral disorders. Overall, this review offers perspectives on the medicinal value of licorice for oral health, opening the door for further research and perhaps even improvements in the treatment of oral diseases.

## 2. Materials and Methods

A search of the literature was conducted using four databases: PubMed, Google Scholar, ScienceDirect, and Web of Science, to find relevant articles on the effects of licorice (*G. glabra*) on oral health and disease. The search was also extended to include reference lists of original and review articles. The search was limited to studies published between 1973 and 2023 in the English language. Unpublished data were not included. Two reviewers assessed the quality and characteristics of the selected studies. The search used a combination of keywords, including “licorice”, “*Glycyrrhiza glabra*”, “oral health”, “oral disease”, and “oral lesions”.

## 3. Chemical and Bioactive Composition of *Glycyrrhiza glabra*

Licorice contains several bioactive compounds that are responsible for its diverse range of pharmacological activities. The major triterpenoid saponin in licorice is glycyrrhizin, which has been extensively studied for its anti-inflammatory, immunomodulatory, hepatoprotective, and antiviral properties [15]. Licorice flavonoids, such as glabridin and isoliquiritigenin (ISL), have also been shown to possess a variety of health benefits, including antioxidant, antimicrobial, and antitumour activities [15]. In this textual description, we have shown some of the key bioactive compounds found in licorice and their associated pharmacological activities [16].

The majority of bioactive substances found in licorice root belong to the chemical classes of triterpenoids and flavonoids. According to several studies, licorice is a good source of proteins, pectins, resins, starches, sterols, gums, simple sugars, polysaccharides, and mineral salts [17]. Phytosterols (sitosterol and stigmasterol), coumarins, vitamins (B1, B2, B3, B5, E, and C), and glycosides are also present in addition to tannins. Along with pantothenic acid, other nutrients that may help in wound healing include lecithin, biotin, niacin, manganese, calcium, calcium salts, proteins, and nucleic acids [3].

### 3.1. Flavonoids and Isoflavonoid

- Glabridin: antioxidant, muscle relaxant.
- Glabrene: antiulcer.
- Isoliquiritigenin (ISL): antimycobacterial, uterine relaxant and analgesic, antitussive activity.
- Licochalcone: anticancer, antimalarial.
- Liquiritigenin: corticosteroid activity, antimicrobial.

### 3.2. Triterpenoid Saponin

- Glycyrrhizic acid (GL): antiulcer, antiallergic, antiviral activity, antihyperglycemic.
- 18-β-glycyrrhetinic acid (GA): memory-enhancing activity, corticosteroid activity, antiviral activity, immuno-stimulating activity.
- Glycyrrhizin: corticosteroid activity, antiallergic, hepatoprotective, anti-inflammatory, antiviral activity, antihyperglycemic, immuno-stimulating activity.

### 3.3. Coumarin

- Licocoumarin: Uterine relaxant and analgesic.

These activities are not exclusive to the chemical components listed, and some activities are listed under different components due to their various potential effects [18].

A summary of the chemical bioactive composition, structure, and function is demonstrated in Table 1.

GL and glabridin can be extracted from licorice using different solvents, such as water, methanol, ethanol, acetonitrile, and chloroform. Some studies have shown that the optimum condition for this extraction would be under 50 °C, and it takes around 60 min [19]. The enzyme-assisted extraction of GL was also used in another study [20]. In a more recent study, a warm mixture of acetone and diluted nitric acid was used for 2 h to extract GL [21].

## 4. Mechanism of Action

The active ingredients of licorice show wide biological activities that can boost the body’s immunity and protect against different diseases. It has antibacterial, antiviral, anti-inflammatory, analgesic, and antioxidant properties, as well as many others [15]. Different pathways are responsible for regulating these actions, which we will discuss briefly.

### 4.1. Antiviral

Glycyrrhizic acid (GL) has been shown to have potential immunomodulatory properties by inhibiting the spread of viruses and disassembling their particles [16]. Its antiviral activity is clearly demonstrated by different studies on the herpes simplex virus, Epstein–Barr virus (EBV), Hepatitis C virus (HCV) [22], and SARS coronavirus [23]. Glycyrrhizin’s antiviral activity is primarily achieved by inhibiting virus entrance, decreasing cell membrane fluidity, and promoting interferon production [23].

### 4.2. Antibacterial/Antifungal

Licorice aqueous extract, ethanol extract, and supercritical fluid extract have powerful inhibitory effects on both Gram-positive and Gram-negative bacteria, including *Staphylococcus aureus*, *Escherichia coli*, *Pseudomonas aeruginosa*, and *Candida albicans (C. albicans).* This occurs due to inhibiting biofilm formation, preventing yeast–hyphal transition, reducing toxin production, and decreasing the production of α-hemolysin [22]. Licochalcone, glabrene, and glabridin have shown antimicrobial action against *Helicobacter pylori* [24].

### 4.3. Anti-Inflammatory

Glycyrrhizin is a well-known anti-inflammatory ingredient that has been shown to enhance the duration of plasma recalcification and to extend thrombin and fibrinogen coagulation time in vitro, making it the first plant-based thrombin inhibitor [16], by reducing prostaglandin E2 (PGE2), matrix metalloproteinases (MMPs), tumour necrosis factor (TNF), and free radicals. Licorice has demonstrated anti-inflammatory properties that are supported by its long-standing applications for easing coughing, removing phlegm, promoting digestion, reducing pain, and a variety of other functions [25].

### 4.4. Immunomodulator

Glycyrrhizin is also capable of activating macrophages. *G. glabra* polysaccharide fractions have shown immuno-stimulating activity, boosting the immune response [16]. Glycyrrhizin selectively enhances Fas-mediated apoptosis in human T cell lines. Moreover, GA can also induce type 2 antagonistic CD41 T cells, as shown in some in vivo and vito studies [24]. Both compounds have the ability to induce interferon activity and inhibit TNF-alpha and IL-8 production [24].

### 4.5. Analgesic

Licorice’s inhibitory effects on the functions of cyclooxygenase-2 (COX-2), high mobility group box 1 protein (HMGB1), and gap junction/connexin raise the possibility that some of the plant’s components may be useful for treating pain [26]. Painful illness can be treated with licorice within a combination of glycyrrhiza uralensis and radix paeoniae alba in equal amounts; this combination is well known to relive myalgia, arthralgia, and neuropathic pain [26].

### 4.6. Antioxidant

Significant antioxidant activity is present in licorice phytochemicals. Licorice prevents neutrophils from producing reactive oxygen species (ROS) at the site of inflammation. The licochalcone B and D found in *G. glabra* exhibit potent 2,2-diphenyl-1-picryl-hydrazyl-hydrate (DPPH) radical scavenging action, as well as the capacity to stop the peroxidation of microsomal lipids [27,28].

### 4.7. Anticancer

By increasing mitochondrial permeability transition, GL has been found to support the proapoptotic pathway, which, in particular, encourages tumour cells to undergo apoptosis [16]. Twelve licorice flavonoids have been found to limit the growth of non-small-cell lung cancer cells by inhibiting the cell cycle at various stages and inducing apoptosis [25,29].

## 5. Effect of Licorice on Oral Health

Because of its many biological advantages, such as its anti-inflammatory, antioxidative, and antibacterial activities, licorice has been extensively researched [15]. This is because antimicrobial drug resistance has necessitated the creation of alternative medications. Numerous clinical trials and animal studies have been carried out globally to assess the potential of licorice and its bioactive components, such as glabridin, licoricidin, licorisoflavan A, licochalcone A, and glycyrrhizin, in the prevention and treatment of several oral diseases such as dental caries, periodontal diseases, candidiasis, aphthous ulcers, and even severe conditions such as oral cancer [18].

### 5.1. Dental Caries

Studies carried out in recent decades have confirmed the anticariogenic role of natural compounds extracted from licorice, specifically glycyrrhizin and triterpenoid saponin glycosides in *G. glabra*. The first property investigated was the antiadherent property of glycyrrhizin that inhibits the glucosyltransferase activity of *Streptococcus mutans*, which is required for biofilm formation [30]. Several studies have investigated the surface coating effect of glycyrrhizin, but conflicting results have been reported [31,32,33]. Other studies have used licorice as an ingredient in oral hygiene products [34], but additional clinical trials are required to confirm its potential effectiveness in controlling dental caries.

### 5.2. Periodontal Disease

The use of herbal formulations is seen as an attractive alternative to conventional antibiotics. Thus, the use of licorice in periodontal therapy has been studied. Licorice extract demonstrated potent anti-inflammatory effects by inhibiting inflammatory cytokines [35,36]. A recent study has shown that licorice and chlorhexidine mouthwash both prevent plaque buildup and gingival irritation [37]. Since the chemical ingredients of mouthwashes commonly available on the market have many side effects with prolonged usage, herbal mouthwash has been a better alternative for self-care treatment. Another study showed that patients who used *G. glabra* gum paint at a 10% concentration experienced a considerable reduction in gingival bleeding, probing pocket depth, and attachment loss [38]. A comprehensive review has documented a list of studies investigating the therapeutic potential of licorice extract in periodontal therapy [28]. As they have no side effects, they can be used for extended periods of time; therefore, they are an effective substitute for chemicals in the prevention and treatment of periodontal disease. On the other hand, limited studies have investigated the effect of licorice on gingivitis. Aqueous extracts of raw polysaccharides from *G. glabra* have been shown to have strong antiadhesive effects against *Porphyromonas gingivalis (P. gingivalis)* [39].

### 5.3. Oral Candidiasis

Several animal studies have been conducted investigating the antifungal effect of licorice compounds. In one study, glycyrrhizin treatment increased mice’s resistance to *C. albicans* infection [40]. The antifungal effects of organic solvent extracts of *G. glabra* against *C. albicans* have previously been reported [41]. In an in vitro investigation, it was observed that glabridin exhibits a significant effect against amphotericin-B-resistant isolates of *C. albicans* [42]. Another study demonstrated that by influencing the CD4+ Th1 immune response, the licorice flavonoid liquiritigenin exhibits an immunomodulatory effect and can protect mice from disseminated candidiasis [43]. Investigations have also been conducted on how licorice ingredients affected the virulence characteristics of *C. albicans*; it was found that glabridin and licochalcone A both blocked the yeast–hyphal transition. Additionally, they claimed that nystatin and each of these substances could work together to combat *C. albicans* [44].

### 5.4. Recurrent Aphthous Ulcer

Various studies have been published on the effectiveness of licorice in reducing aphthous ulcers’ healing time and controlling pain. Using a mouthwash with a deglycyrrhizinated licorice extract for two weeks seemed to reduce discomfort and hasten the healing of aphthous ulcers [45]. The efficacy of licorice bioadhesive hydrogel patches to promote healing and relieve pain suggested that mechanical mucosal protection alone was crucial in lowering pain and encouraging healing. This conclusion was reached as licorice-containing biopatches are nearly as effective as the control patches without licorice. Due to the small sample size and the use of a low licorice concentration (1%) in the experiment, this study was not conclusive [46]. On the other hand, using a dissolving oral patch containing licorice extract for up to 8 days reduced ulcer size and pain compared to using a placebo patch in a randomised, double-blind clinical trial [47]. It was also shown that over-the-counter patches containing licorice extract modify the course of aphthous ulcers by shortening the time and reducing the size and discomfort of lesions, which speeds up healing [48].

### 5.5. Herpes Simplex Virus Infection

Due to its antiviral activity, the potential use of licorice in reducing the severity and duration of herpes simplex virus (HSV) infections has been investigated. It has been demonstrated that by creating an environment that was resistant to HSV1 replication, glycyrrhizin possessed its anti-HSV1 action [49]. A clinical trial has demonstrated that the topical application of the GA derivative (Carbenoxolone) in the management of herpetic gingivostomatitis and recurrent herpes labialis was very effective in reducing the pain and healing time [50]. Another study has tested glycyrrhizin gel on patients who suffer from herpes on their lips and nose; this study also showed a reduction in pain and healing time as in the previous study [51]. In addition, a more recent study evaluated the design and formulation of a gel containing extracts of five herbs, including licorice, and concluded that it reduced the recovery time and that the anti-inflammatory, local analgesic, and wound-healing properties are attributed to licorice and rosemary [52]. Furthermore, a successful resolution upon using topical botanical gel treatment for recurrent oro-facial herpes simplex was reported in a single case [53].

### 5.6. Xerostomia

The effect of licorice on xerostomia studied in haemodialysis patients in a randomised controlled trial (RCT) has shown that only the licorice mouthwash offered subjective xerostomia alleviation. Clean water and licorice-containing mouthwash have both improved the objective measurement of salivary flow rate [54]. This implies that using licorice mouthwash may help haemodialysis patients who have dry mouth. It has been suggested that since licorice has a sweet flavour and acts as a gustatory stimulant, it may stimulate saliva production and improve the symptom of xerostomia, which was noted among haemodialysis patients [55].

### 5.7. Oral Lichen Planus (OLP)

A Japanese study has shown that patients with OLP who tested positive for HCV have shown clinical improvement in OLP after the intravenous administration of glycyrrhizin [56,57]. Intravenous glycyrrhizin therapy for healthcare patients is well established; it is unclear if this treatment has its effect directly on lichen planus lesions or the underlying viral hepatitis. It would be much easier to understand this mechanism if the topical application of licorice extract was utilized. However, it has been demonstrated that licorice patches are effective at reducing pain but not at improving clinical symptoms compared to topical steroid therapy [58]. Meanwhile, a reduction in pain, erythema, burning sensation, and functional disturbance after using a prepared mouthwash formulation of aloe vera, licorice, and sesame oil in patients with OLP has been reported [59].

### 5.8. Halitosis

It has been shown that licorice extract and the two isolates, licoricidin and licorisoflavan, can serve as natural components that have the ability to decrease the production of bacterial volatile sulphur compounds by *P. gingivalis*, *Prevotella intermedia*, and *Solobacterium moorei* and thus potentially manage halitosis [60]. Compared to conventional mouthwashes, the ability of Manuka honey and licorice root extract to reduce *P. gingivalis* bacterial growth has been studied. There are some safety concerns about the available commercial mouthwashes, necessitating the search for more natural ingredients [61].

### 5.9. Oral Mucositis

In cancer patients, particularly those with head and neck cancer, *G. glabra* extract can be used effectively in the prevention and treatment of oral mucositis following radiation and chemotherapy. It is advantageous in two ways: first, there are no interruptions of their chemotherapy or radiation; second, their food intake is not significantly impacted, allowing patients to maintain their nutritional state [62]. In an RCT, it has been reported that aqueous glycyrrhiza extract can be useful for reducing the severity of oral mucositis in patients with head and neck cancer receiving radiotherapy compared to a placebo [63]. Another RCT found that both triamcinolone and licorice mucoadhesive films are effective in the management of oral mucositis during radiotherapy [64]. A clinically significant decrease in mucositis was also observed when lyophilised licorice extract (5% *w*/*v*) was used as a mouthwash before and right after each session of radiation therapy [65].

### 5.10. Oral Squamous Cell Carcinoma (OSCC)

Isoliquiritigenin (ISL) is a flavonoid with a significant therapeutic potential for treating adenoid cystic carcinoma and the potential to be used as a cancer chemotherapeutic agent [66]. In another study, licochalcone A was reported to induce the apoptotic cell death of OSCC cells; thus, it can be used as a chemotherapeutic agent [67]. Further cell line studies have reported similar findings [68,69,70]. In addition, it was found that human oral cancer cell proliferation was inhibited by a polysaccharide found in *Glycyrrhiza inflata* by causing apoptosis via the mitochondrial pathway [71].

### 5.11. Oral Submucous Fibrosis (OSF)

Licorice was suggested as a treatment for OSF because it demonstrated antifibrotic effectiveness in human fibroblast cell lines [72].

### 5.12. Implications in Endodontics

The usefulness of licorice as a root canal irrigant and medication has only been studied in a small number of in vitro investigations. In one of these studies, it was demonstrated that, compared to Ca(OH)_2_ alone, licorice extract had a much greater inhibitory impact against *Enterococcus faecalis* (*E. faecalis*) [73]. Furthermore, another study has shown that with zinc oxide eugenol-based sealer, the highest zones of bacterial growth inhibition were seen when *G. glabra* was added [74]. A recent study has found that propolis (a resin-like material made by bees from the buds of poplar and cone-bearing trees) is more effective against *E. faecalis* compared to *G. glabra* and Ca(OH)_2_, despite the reduction in the number of colonies in all types of irrigation [75].

**Figure 1:**
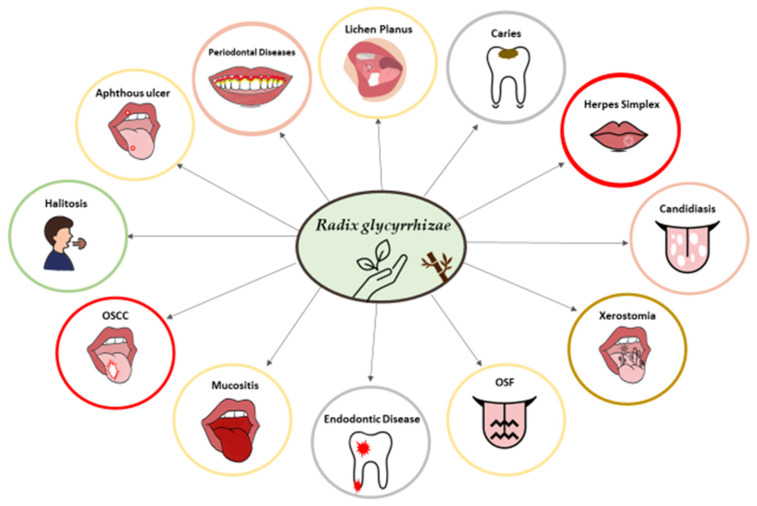
Effect of licorice (*Radix glycyrrhizae*) on oral health.

An illustration of the effects of licorice on oral health is shown in Figure 1.

## 6. Dosage and Concentration

In the past, in the United States, the production of several dietary supplements, including licorice, was not strictly controlled. A 100 mg/day maximum limit for glycyrrhizin consumption was suggested by the European Union based on investigations with human volunteers. The Dutch Nutrition Information Bureau warns against exceeding a daily glycyrrhizin intake of 200 mg, or 150 g of licorice candy. Licorice fluid extracts have a glycyrrhizin content of 10–20% and typically include 200–800 mg. About 2% of frequent users ingest more GA than the recommended daily limit of 100 mg [76].

For most applications, a high level of GL in the blood with fewer side effects is recommended. A dosage of 200 mg powder of deglycyrrhiznated licorice (DGL) dissolved in 200 mL of warm water used as mouthwash four times daily has been suggested [77]. Glycyrrhizin-containing products can also be used intravenously to treat different viral infections, such as HCV, in which 0.2% of glycyrrhizin is used as part of Stronger Neo-Minophagen C (SNMC) saline solution. It can be used in a topical gel of 2% GL to treat some skin and oral conditions, such as eczema or herpes simplex infection. Generally, the following doses taken three times a day are considered to be safe and can effectively raise GL levels: 1–2 g of powdered root, 2–4 mL of fluid extract (1:1), and 250–500 mg of solid dry powder extract (4:1) [77].

## 7. Overview of the Potential Adverse Effects of *Glycyrrhiza glabra*

Although licorice root is a natural, bushy herb that is frequently used as a flavouring agent in food and beverages as well as for therapeutic purposes, it can have certain systemic negative effects when ingested in high quantities or used for extended periods of time. The Food and Drug Administration (FDA) has designated licorice as “Generally Recognised as Safe” if used carefully by those who are not allergic to glycyrrhizin. Licorice consumption in excess can result in hypertension, hypokalaemia, rhabdomyolysis, respiratory problems, muscle paralysis, hyperparathyroidism, abrupt renal failure, and encephalopathy. The World Health Organisation (WHO) states that licorice at a dose of 100 mg per day can be consumed without risk. Due to the antiplatelet and anticoagulant properties of licorice, individuals who take anticoagulants for cardiovascular or cerebrovascular illnesses along with herbal treatments containing licorice may experience a risk of excessive bleeding [28]. Glycyrrhizin, the primary active component of licorice root, can result in the following systemic adverse effects.

### 7.1. High Blood Pressure

Licorice raises blood pressure by inhibiting the 11-beta-hydroxysteroid dehydrogenase enzyme. This enzyme is involved in metabolising the hormone cortisol, which controls blood pressure. When licorice blocks this enzyme, the body’s cortisol levels rise, which causes sodium and water retention; as a result, blood pressure is increased [78].

### 7.2. Hypokalaemia (Loss of Potassium)

Licorice can make the body lose potassium, which is needed for healthy neuron and muscle function. The increased activity of the hormone aldosterone, which controls the body’s sodium and potassium levels, is the reason for potassium loss. As licorice prevents aldosterone from being broken down, sodium and water are retained, raising blood pressure, while potassium is expelled in urine [79,80].

### 7.3. Unbalanced Hormones

Licorice can impair the adrenal gland’s ability to produce hormones like cortisol and aldosterone. Men’s testosterone levels may drop as a result of this interference, which could result in symptoms including weariness, erectile dysfunction, and a reduced libido. Licorice can raise oestrogen levels in women, which can result in mood swings, sensitive breasts, and irregular menstruation [81].

### 7.4. Liver Damage

Glycyrrhizin, the key ingredient in licorice, can harm the liver if ingested in significant quantities over an extended period of time. Although it is uncommon, this effect can happen in people who ingest significant doses of licorice supplements or teas [25].

Other side effects were associated with gastrointestinal, skin and subcutaneous tissue, and hepatobiliary disorders [82]. It is important to note that the majority of these systemic negative effects are linked to long-term, heavy licorice use. In general, the occasional use of licorice supplements or eating moderate amounts of it as a food-flavouring agent is unlikely to affect an individual significantly. To prevent any interactions or negative effects, it is crucial to speak with a healthcare professional before consuming licorice if you have a pre-existing medical condition or are using any medication [25,27].

## 8. Dental Side Effects

When used frequently or in excessive doses, licorice might have certain unfavourable dental effects. The licorice ingredient glycyrrhizin in particular might result in the following dental problems:

### 8.1. Tooth Decay and Enamel Erosion

When taken in its natural state, licorice root can offer certain advantages for dental health. However, licorice candies and other licorice-flavoured goods that are high in sugar can contribute to dental decay. Sugar is metabolised by oral bacteria to create acid, which can erode tooth enamel and cause cavities [83].

### 8.2. Discoloration

Over time, especially if ingested in large quantities, licorice sweets and other dark-coloured foods can discolour the teeth and tongue temporarily and result in a brownish appearance. This discolouration could last longer and appear darker if licorice is used in combination with tobacco products [84].

If one routinely ingests licorice, it is crucial to take precautions to preserve their dental health. This can involve using fluoride toothpaste, brushing and flossing often, and going to the dentist for routine examinations. The acid produced can be neutralised, and food residues can be washed away by drinking water [85].

## 9. Contraindications

Licorice should not be used by patients who have uncontrolled hypertension. Additionally, the primary active ingredient in the root, GA, can cause what seems to be pseudohypoaldosteronism [86]. This causes a person to become hypersensitive to the hormones produced by the adrenal cortex, which may lead to a number of side effects such as heart attacks, migraines, high blood pressure, weariness, and water retention, sometimes causing leg oedema and other complications [86]. It is contraindicated to use licorice in patients who are under corticosteroids or other drugs that can also deplete potassium [87]. Several studies have highlighted the potential negative effects of licorice consumption on the cardiovascular system, particularly in relation to blood pressure regulation [78,88,89].

Deutch and colleagues have conducted a comprehensive review summarising recent research in this area [78]. Similarly, Penninkilampi has found comparable conclusions regarding the chronic ingestion of licorice and hypertension [88]. Additionally, another study by Nazari has reported the moderate toxicity of licorice and glycyrrhizin salts, with significant adverse effects including secondary hypertension and hypokalaemia [89]. Glycyrrhizin is contraindicated for use with oral contraceptives and should be used with caution during pregnancy [16].

## 10. Interaction with Other Plants or Medications

The pharmacokinetics and pharmacodynamics of many drugs may vary when taken simultaneously with glycyrrhizin or GA. In general, due to their potential drug interactions, GZ, GA, or related drugs should be used with caution when combined with other medications [90]. It has also been found that licorice and its phytochemicals may influence the metabolism and clearance of specific medications if they are substrates for CYP3A4 and CYP1A2 (two major cytochrome P450 isoforms) [91]. Additionally, glycyrrhizin can affect some drug transporters by reducing or inhibiting their activity. This will interfere with the absorption, distribution, metabolism, and excretion of these drugs that are substrates for these transporters [92]. Licorice should not be used with Sargassum (Hai Zao), Herba Cirsii Japonici (Da Ji), *Euphorbia kansui* (Gan Sui), or Flos genkwa (Yuan Hua), according to the theories of traditional Chinese medicine. These four plants, taken along with licorice, may weaken liver function and cause cardiac toxicity [93].

## 11. The Availability of Different Forms of Licorice Products in the Kingdom of Saudi Arabia

### 11.1. Forms

- Licorice root powder [94];
- Licorice root extract [80];
- Licorice lozenges [95];
- Licorice mouthwash [96];
- Licorice tea [97].

It is necessary to keep in mind that regional restrictions and the availability of particular licorice products could vary. For advice on where to find licorice goods in the KSA, it is usually preferable to speak with a medical expert or a nearby merchant. It is also crucial to make sure that any licorice items you buy are safe to eat and from reliable suppliers.

### 11.2. Availability

The availability of licorice products in the KSA may vary depending on the region and local regulations. However, listed below are a few places where you may be able to find licorice products:

- Health food stores (Atara Shops): some health food stores in the KSA may carry licorice root powder, licorice root extract, licorice lozenges, and other licorice products.
- Pharmacies: licorice root extract, licorice lozenges, and licorice mouthwashes may be available at pharmacies in the KSA.
- Supermarkets: licorice tea may be available at some supermarkets in the KSA, particularly in the health food or tea sections.
- Online retailers: there are several online retailers that sell licorice products, including licorice root powder, licorice root extract, licorice lozenges, licorice mouthwash, and licorice tea, that are local or can be shipped to the KSA.

## 12. Conclusions

In conclusion, licorice possesses numerous biological advantages, resulting in extensive research on its potential therapeutic applications. Studies have investigated the use of licorice and its bioactive components in the prevention and treatment of different oral diseases. Licorice has shown anticariogenic effects, anti-inflammatory effects, and the ability to reduce discomfort and promote healing in some oral conditions when used within mouthwashes or as bio-adhesive patches. Overall, licorice holds promise and power as a natural therapeutic agent for various oral diseases. However, more clinical trials and research are necessary to validate its effectiveness, explore its full therapeutic potential, and establish standardised treatment protocols. These studies may encourage different pharmaceutical companies to manufacture a wider range of licorice forms. Undoubtedly, this should be conducted in a proper, well-controlled manner.

## Figures and Tables

**Table 1:** Chemical and bioactive composition of *Glycyrrhiza glabra* and their functions.

Chemical Components	Structure	Pharmacological Action
Flavonoids and Isoflavonoid		
Glabridin	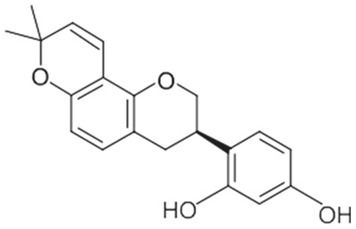	Antioxidant Muscle relaxant
Glabrene	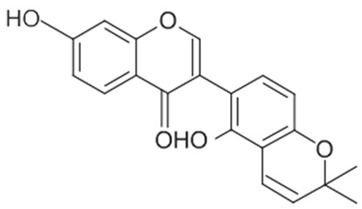	Antiulcer
IsolIsoliquiritigenin (ISL)	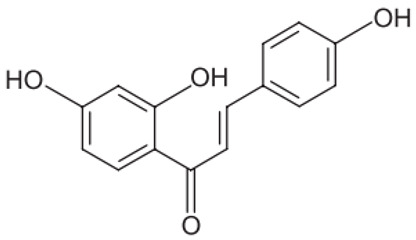	Antimycobacterial, Uterine relaxant Analgesic, antitussive activity Corticosteroid activity Antimicrobial
Licochalcone	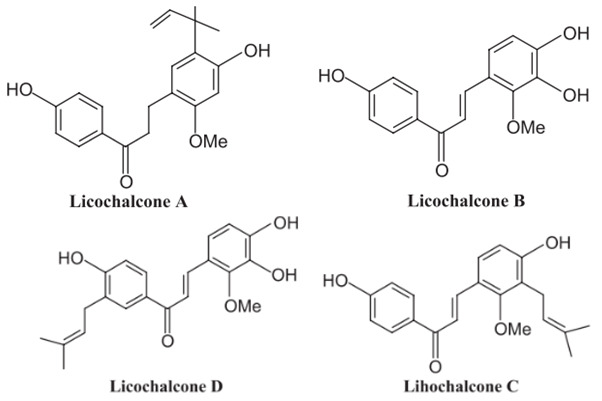	Anticancer Antimalarial
Triterpenoid saponin		
Glycyrrhizic acid (GL)	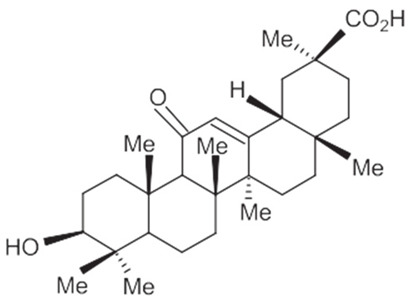	Antiulcer Antiallergic Antiviral activity Antihyperglycemic
18-β-glycyrrhetinic acid (GA)	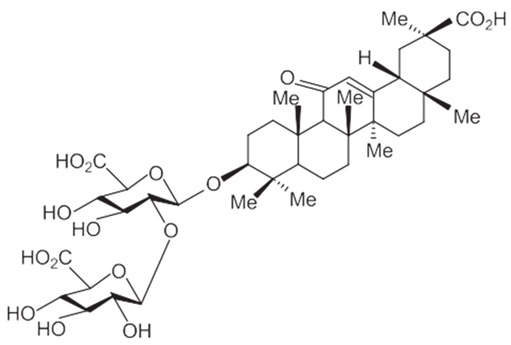	Memory-enhancing activity Corticosteroid activity Antiviral activity Immunostimulating activity
Glycyrrhizin	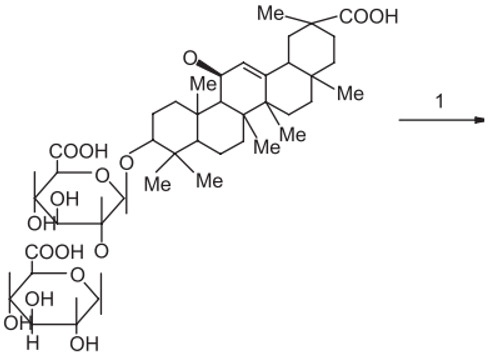	Corticosteroid activity Antiallergic Hepatoprotective Anti-inflammatory Antiviral activity Antihyperglycemic Immunostimulating activity
Coumarin		
Licocoumarin	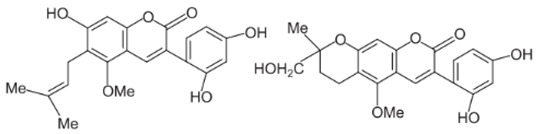	Uterine relaxant Analgesic

## Data Availability

Data are available upon request from the authors (haldehlawi@kau.edu.sa).

## Abbreviations

*C. albican*	*Candida albicans*
COX-2	Cyclooxygenase-2
DGL	Deglycyrrhiznated licorice
DPPH	2,2-diphenyl-1-picryl-hydrazyl-hydrate
EBV	Epstein-Barr virus
*E. faecalis*	*Enterococcus faecalis*
FDA	Food and Drug Administration
GA	18-β-glycyrrhetinic acid
*G. glabra*	*Glycyrrhiza glabra* L.
GL	Glycyrrhizic acid
HCV	Hepatitis C virus
HMGBI	High mobility group box 1 protein
HSV	Herpes simplex virus
ISL	Isoliquiritigenin
KSA	Kingdom od Saudi Arabia
MMPs	Matrix metalloproteinases
OLP	Oral lichen planus
OSCC	Oral Squamous Cell Carcinoma
OSF	Oral submucous fibrosis
*P. gingivalis*	*Porphyromonas gingivalis*
PGE2	Prostaglandin E2
RCT	Randomised controlled trial
ROS	Reactive oxygen species
SNMC	Stronger Neo-Minophagen C
TNF	Tumour necrosis factor
WHO	World Health Organisation
